# Analysis of quality raw data of second generation sequencers with Quality Assessment Software

**DOI:** 10.1186/1756-0500-4-130

**Published:** 2011-04-18

**Authors:** Rommel TJ Ramos, Adriana R Carneiro, Jan Baumbach, Vasco Azevedo, Maria PC Schneider, Artur Silva

**Affiliations:** 1 Instituto de Ciências Biológicas, Universidade Federal do Pará, Belém-PA, Brazil; 2 Instituto de Ciências Biológicas, Universidade Federal de Minas Gerais, Belo Horizonte-MG, Brazil; 3 Institute for Genome Research and Systems Biology. Center for Biotechnology. Germany Institute for Genome Research, Bielefeld University, Bielefeld, Germany

## Abstract

**Background:**

Second generation technologies have advantages over Sanger; however, they have resulted in new challenges for the genome construction process, especially because of the small size of the reads, despite the high degree of coverage. Independent of the program chosen for the construction process, DNA sequences are superimposed, based on identity, to extend the reads, generating contigs; mismatches indicate a lack of homology and are not included. This process improves our confidence in the sequences that are generated.

**Findings:**

We developed Quality Assessment Software, with which one can review graphs showing the distribution of quality values from the sequencing reads. This software allow us to adopt more stringent quality standards for sequence data, based on quality-graph analysis and estimated coverage after applying the quality filter, providing acceptable sequence coverage for genome construction from short reads.

**Conclusions:**

Quality filtering is a fundamental step in the process of constructing genomes, as it reduces the frequency of incorrect alignments that are caused by measuring errors, which can occur during the construction process due to the size of the reads, provoking misassemblies. Application of quality filters to sequence data, using the software Quality Assessment, along with graphing analyses, provided greater precision in the definition of cutoff parameters, which increased the accuracy of genome construction.

## Background

The introduction of second-generation genome sequencing has reduced the cost and time required for genome construction; this method generates large amounts of data and increased sequencing coverage when compared to the dideoxy terminal Sanger method [1]. However, this new methodology reduces the size of the readings and has brought challenges to the genome assembly process, such as a need to develop efficient algorithms to reconstruct the genome [2]. Several examples of programs suitable for genome assembly from short reads are Velvet [3], Edena [4], SHARCGS [5], VCAKE [6], ALLPATHS [7], Euler-SR [8], and Quality-value guided Short Read Assembler (QSRA) [9]. All of them involve a process of connecting overlapping DNA sequences; however, only QRSA considers the quality of the reads during the assembly process.

Regardless of the assembly method used, data preparation is necessary. One step in this preparation is the quality filter, whenever readings are taken with a lower phred quality [10]. Independent of the genome construction system, it is necessary to prepare the data. One of the steps in data preparation is a quality filter, with which reads with low phred quality are removed. This improves the alignment of the sequences to avoid problems due to mismatches [11]. Li et al. (2010) observed a 50% decrease in alignment errors when bases screened for quality were used; this is an important part of the preparation required for producing accurate results.

The cutoff value for read quality affects the coverage and especially the quality of sequencing. Very stringent parameters can reduce the coverage of the genome and hinder the assembly process. Also, using poor-quality bases that are products of mismatches can lead to less accurate results. To address this problem, we developed the software Quality Assessment (QA), with which one can review graphs showing the distribution of quality values from the sequencing reads, including the average quality, and the accumulated quality for each of the bases; this information can be used to estimate the coverage and quantity of the readings that pass through the quality filter.

### Input format

QA receives two files as input: the first with standard-only Phred quality values for each base of a read, and the second containing the sequences in nucleotides or color space (SOLiD). The input files must have equal size sequences such as those generated by the SOLiD and Illumina platforms in order to be used for the generation of quality graphs.

### Sample Data

The data that we tested with this software were obtained from sequencing of *Corynebacterium pseudotuberculosis *(Cp162) and *Exiguobacterium antarcticum *(B7) with SOLiD system, using a library of fragments with readings of 35 base pairs (bp) and a mate-pair library with 25 bp for each tag, F3 and R3, respectively [12]. We obtained 21,102,241 readings from the Cp162 data, and 44,171,676 and 45,024,226 readings, from the B7 tags F3 and R3, respectively.

The estimated genome coverage was obtained using the formula *C *= (n * *L*)/*S*, where *C *is the estimated coverage, n is the number of readings, *L *is the size of the reads and *S *is the expected size of the genome [13]. The expected sizes for the genomes used in this study were defined based on phylogenetically-related organisms deposited in Genbank. For Cp162, a size of 2.3 mega bases (Mb) was obtained based on *Corynebacterium pseudotuberculosis *FRC41 (CP002097), and for B7, about 3 Mb was obtained based on *Exiguobacterium sibiricum 255-15 *(CP001022).

### Implementation

**Figure 1:**
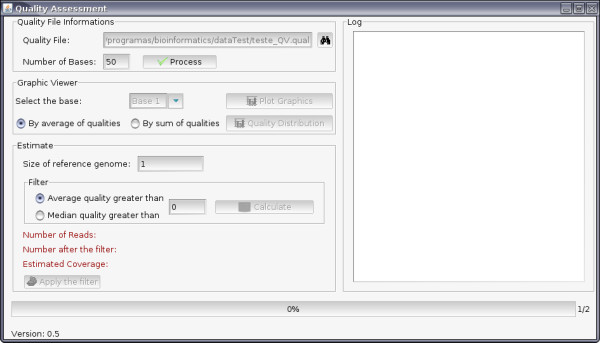
**Main screen of the Quality Assessment software**. The quality input file and the size of the expected reads must be defined to start the data process. After it, the quality graphs can be generated using the specific buttons.

The software was developed in JAVA programming language http://java.sun.com/, using the paradigm of object orientation and the graph library Swing http://java.sun.com/docs/books/tutorial/uiswing. Input is raw files from the sequencing machine (multifasta format): (i) files containing the quality values of phred for the readings [14] and (ii) sequences in color space [15] or nucleotide format; this information is solicited only at the time that the quality filter is applied to the data. The software (Figure 1) offers an option in which the size of the expected reads is informed, and when processing is finished it generates a log that shows the multifasta-file-sequence formatting problems: invalid characters, blank lines, and reads that are not of the expected size; these are eliminated in the processing. Optionally, the software can be run without the graphing interface; however, in this option it is not possible to estimate the coverage of the genome, and the phred quality values need to be previously defined.

The raw data file, which includes information on the quality of the sequences, is processed, and the frequencies of the mean and median values for each base are stored in a *hash *table, to be used to calculate the estimated coverage of the sequencing and for the generation of the graphs that show the distribution of the base quality values and means, using the library *JfreeChart *http://www.jfree.org/jfreechart/.

Applying the filter to the raw data files requires a large memory; for this reason, after the first file is generated, the memory reserved for the execution of the process is liberated to the operational system through the Java language resource know as garbage collection, run by the program itself. The filtered files are stored in the same original directories, with the extension.*new *added to each file name.

## Results and Discussion

**Figure 2:**
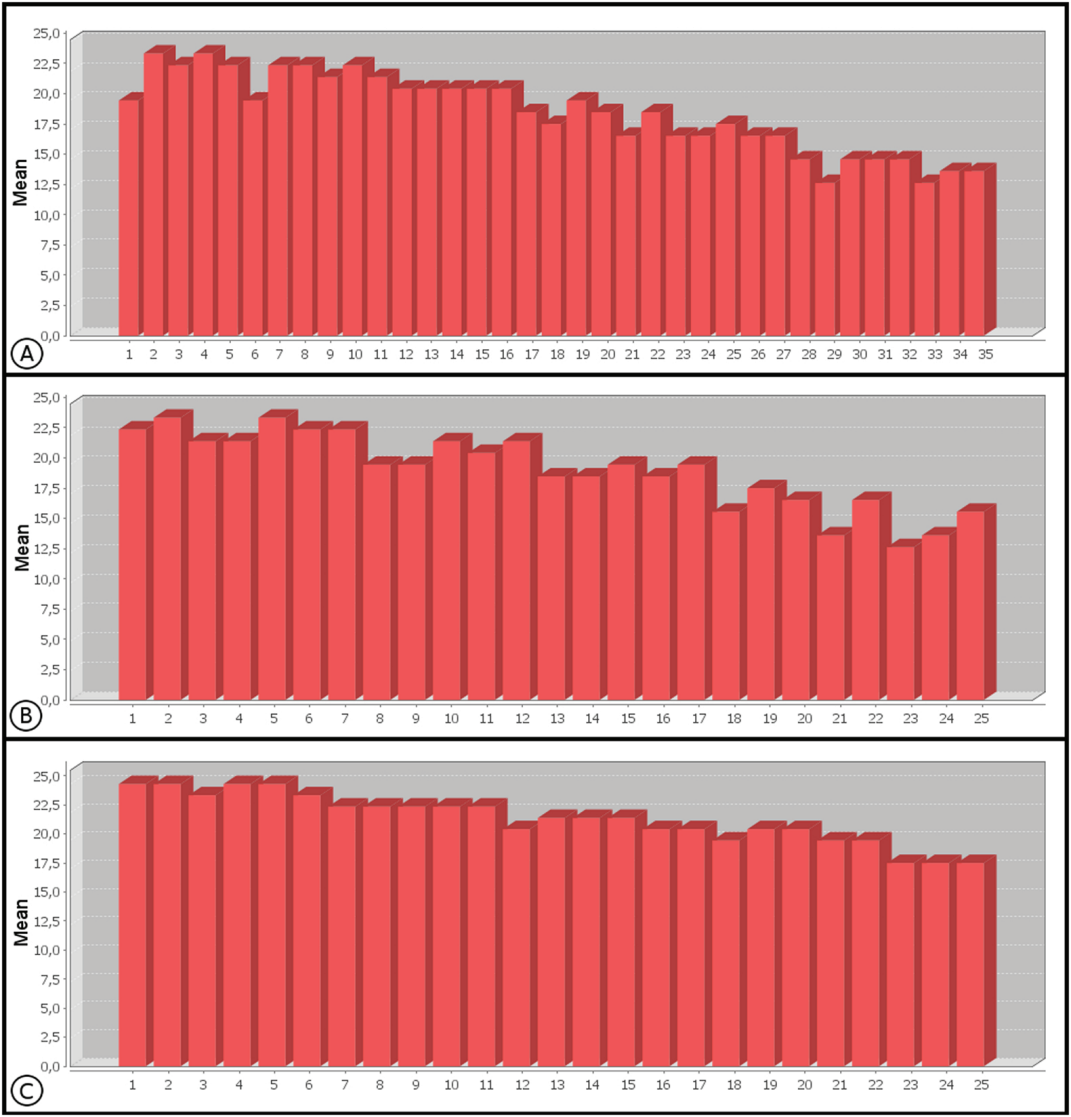
**Evaluation of mean quality per base in the sequence**. Graphic representation of the mean quality observed per base of sequenced raw data of Cp162, B7 (F3), and B7 (R3) as a plot of the base position (X-axis) against mean base quality (Y-axis). A: Cp162 data containing 35 bp; B7(F3) data containing 25 bp; C: B7(R3) data containing 25 bp.

**Figure 3:**
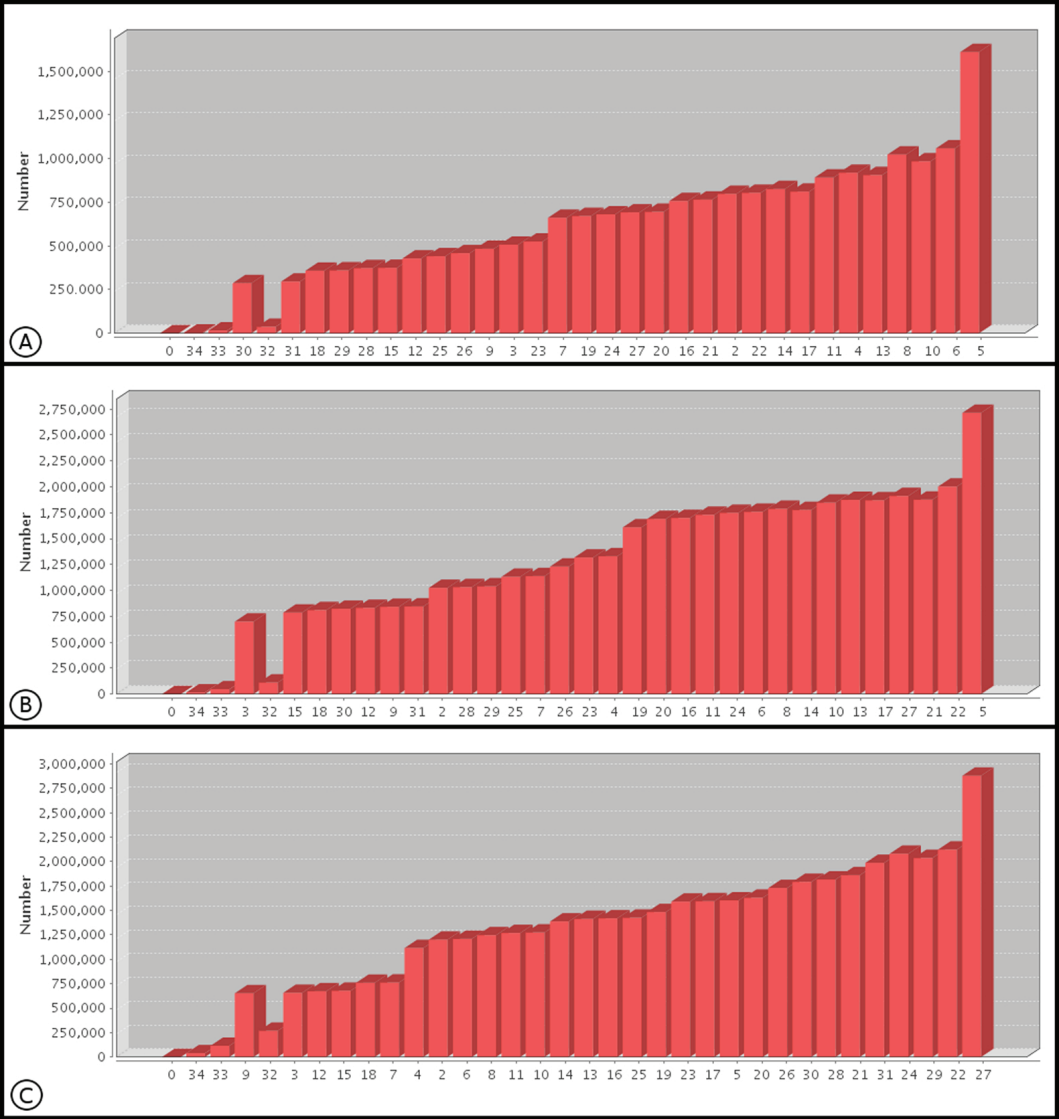
**Frequencies of quality values for the last base of the reads**. Distribution of the Phred quality of the last base of raw data reads of Cp162, B7(F3) and B7(R3) as a plot of the observed Phred quality value (X-axis) against frequency of occurrence. A: Cp162; B: B7 (F3); C:B7 (R3).

The mean quality of each of the 35 sequence bases from the Cp162 data can be observed in Figure 2a; 17 of these gave a mean quality equal to or greater than phred 20, while the terminal bases of the reads had a mean quality of less than 20 [16]. Figure 3a shows the frequency of the quality values of the 35^th ^base of Cp162, with phred 5 being the most common value, which influences the reduction in mean quality observed for this base in Figure 2a. When a cut off filter of phred 20 was applied, the number of reads was reduced by about 43% (Table 1), resulting in a sequence coverage of 172×. Based on the data in Table 2, application of a filter with phred 23 values would give sequence coverage above 100× and a high degree of accuracy of the reads, which would reduce the possibility of misassemblies [17].

**Table 1:** Results of applying the Phred quality filter

	Raw data	Filter by Mean	Filter by Median
**Cp162**	21,102,241	11,349,208(53.78%)	13,140,825(62.27%)
**B7(F3)**	44,171,676	24,927,365(56.46%)	28,216,025(63.87%)
**B7(R3)**	45,024,226	31,809,614(70.64%)	33,765,330 (74.99%)

**Cp162- ***Corynebacterium pseudotuberculosis *strain (fragment library).

**B7 **- *Exiguobacterium antarticum *strain (mate-paired library).

**R3 e F3 **- Reads generated by mate-paired library.

Number of reads generated during the sequencing of Cp162 and B7 (tag F3 and R3) taking into account both the raw data and filtered data, based on the average and median Phred quality, whose cutoff was taken to be 20.

**Table 2:** Genome coverage analysis for different Phred quality values

	Cp162			B7(F3)			B7(R3)		
**Quality Filter**	**QV 20**	**QV23**	**QV25**	**QV20**	**QV23**	**QV25**	**QV20**	**QV23**	**QV25**
	
Mean	172×	104×	52×	207×	134×	76×	265×	217×	172×
Median	199×	152×	116×	235×	182×	142×	281×	248×	220×

Genomic sequencing coverage of Cp162 and B7 (tag F3 and R3) for different Phred quality value cutoffs based on the mean and median.

In the case of IB7 with the tags F3 and R3, both with 25 bp, the mean quality of the bases was above 20, except for the terminal bases shown in Figures 2b and 2c, with tag R3 presenting better quality than F3, except at the 12^th ^base. The terminal bases, Figures 3b and 3c, had the highest frequencies of quality levels at phred 5 and 26 for F3 and R3, respectively, which allows more stringent filters to be applied to tag R3, without excessive loss of coverage, when compared to F3 (Table 2). After applying the quality filter to F3 and R3, with a cutoff at phred 20, the percentage reads discarded for F3 was greater than for R3 (Table 1).

Defining quality values above phred 20 reduced the coverage of the sequences, but it increased data quality, as can be seen in Table 2, in which phred values of 23 and 25 were used as quality cutoff values.

To apply the filter, one can use mean or median quality values observed in the read. When the mean is used, low quality bases can provoke elimination of the read, which can reduce the coverage of the sequencing, though it will also increase the quality [17]. Variation in the quality values of the bases does not influence the median, which could result in a tendency to accept low quality bases, increasing the probability of errors in the genome construction process.

**Figure 4:**
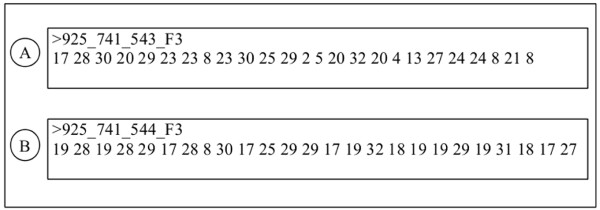
**Fasta sequences with phred quality values**. Example of two Fasta format sequences containing Phred quality values of 25 bp-long reads. A - Fasta sequence with a median and mean Phred qualities of 23 and 19, respectively. B - Fasta sequence with a median and mean Phred qualities of 19 and 22, respectively.

In Figure 4a, we can see a reading with a median value of 23 and a mean of 19; consequently, if we apply a quality filter with phred 20 based on the median, the read would be considered as having six bases with quality below 10. In Figure 4b, if the same filter were applied, the read would be discarded, even though it has a mean quality value of 22.

## Conclusions

Applying a quality filter to raw sequencing data is required in order to reduce sequence construction error, given that the methodologies available for constructing genomes are based on sequence alignment, in which a wrong base can cause a mismatch, making alignment impossible.

The software Quality Assessment allows the operator to visualize quality graphs of the bases in the reads and estimate the coverage based on means or medians, making it possible to select more precise cutoff parameters, reducing the possibility of eliminating high-quality reads or including low-quality reads, which increases the accuracy of the process of constructing genomes from second-generation sequencers.

## Availability and Requirements

**Project name: **QA - Quality Assessment

**Project home page: **http://qualevaluato.sourceforge.net

**Operating system(s): **Platform independent

**Programming language: **Java

**Other requirements: **Java JDK 1.6 or higher

**License: **GNU GPL

Restrictions for use: Permission must be obtained from the author for non-academic/non-public use.

## Competing interests

The authors declare that they have no competing interests.

## Authors' contributions

RTJR developed the program, made the performance tests and contributed to data analysis. ARC tested the program and contributed to the analysis of the results. JB provided critical advice on analysis outputs. MPCS, VA and AS coordinated the work and finalized the manuscript. All authors read and approved the final manuscript.
